# Impaired cortical fissure development in severe fetal growth restriction and correlation with cerebral hemodynamic adaptation: a prospective case–control study

**DOI:** 10.1186/s12884-026-09619-8

**Published:** 2026-07-09

**Authors:** Büşra Tsakır, Gül Alkan Bülbül, Elif Ayan Avcı, Hasan Berkan Sayal, And Yavuz

**Affiliations:** Department of Perinatology, Antalya Training and Research Hospital, Antalya, Turkey

**Keywords:** Fetal growth restriction, Neurosonography, Cortical sulcation, Sylvian fissure, Doppler ultrasonography, Brain development

## Abstract

**Background:**

This study aimed to evaluate cortical maturation and fissure development using fetal neurosonography in fetuses with severe fetal growth restriction (FGR) and to investigate the relationship between cerebral hemodynamic adaptation, assessed by middle cerebral artery pulsatility index (MCA PI), cerebroplacental ratio (CPR) and fetal brain morphology.

**Methods:**

This prospective case–control study included 164 singleton pregnancies (*n* = 82 per group) between 29 and 34 weeks of gestation, comprising 82 fetuses with severe FGR (estimated fetal weight below the 3rd percentile) and 82 gestational age–matched healthy controls. Neurosonographic evaluation included measurements of Sylvian fissure, parieto-occipital fissure, and calcarine fissure depths, as well as corpus callosum length and cerebellar vermian dimensions. Doppler parameters, including umbilical artery and MCA PI, CPR were recorded. Correlations between MCA PI, CPR and neurosonographic measurements were analyzed within the FGR group.

**Results:**

Fetuses with FGR demonstrated significantly reduced Sylvian fissure depth (10.32 ± 1.71 mm vs. 11.79 ± 1.95 mm; *p* < 0.001) and calcarine fissure depth (8.07 ± 1.38 mm vs. 8.44 ± 1.24 mm; *p* = 0.043) compared with controls. No significant differences were observed in corpus callosum length, cerebellar vermian dimensions, or parieto-occipital fissure depth. Within the FGR group, significant inverse correlations were identified between MCA PI and calcarine fissure depth (*r* = − 0.415; *p* < 0.001), corpus callosum length (*r* = − 0.343; *p* = 0.003), vermian length (*r* = − 0.333; *p* = 0.004), and vermian anteroposterior depth (*r* = − 0.261; *p* = 0.027).

**Conclusion:**

Our findings demonstrate that severe FGR is associated with delayed prenatal development of the Sylvian and calcarine fissures. Moreover, the observed inverse correlations between MCA PI values and selected neuroanatomical parameters may reflect region-specific cerebral hemodynamic adaptation and cortical remodeling associated with chronic placental insufficiency, rather than true accelerated or preserved maturation.

## Background

Fetal growth restriction (FGR) is defined as the failure of a fetus to achieve its genetically determined growth potential and represents one of the major challenges in obstetric practice. Affecting 3–10% of pregnancies, this condition is among the leading causes of perinatal morbidity and mortality worldwide [1]. While the adverse perinatal outcomes of FGR have long been recognized, its long-term neurodevelopmental effects have gained increasing importance in recent years. Delays in cognitive, motor, and language development during early childhood have been reported in infants with FGR, and these neurodevelopmental impairments have been shown to persist into school age and adulthood [2, 3].

The primary pathophysiological mechanism underlying FGR is uteroplacental circulatory dysfunction and resulting chronic fetal hypoxemia. Doppler assessments revealing increased resistance in the uterine and umbilical arteries, along with a decreased pulsatility index (PI) in the middle cerebral artery (MCA), indicate that the fetus develops a “brain-sparing” adaptation to this condition [4]. However, it has been demonstrated that this hemodynamic adaptation cannot fully protect cortical development and may lead to alterations in cortical maturation and brain morphology [5].

Magnetic resonance imaging (MRI) studies have reported decreased brain volume, impaired cortical development, alterations in corpus callosum morphology, and differences in connectivity in FGR fetuses [6, 7]. However, widespread clinical use of MRI is limited due to its high cost, time-consuming nature, and restricted accessibility [8]. With advances in imaging technologies, fetal neurosonography has emerged as a more practical, accessible, and cost-effective method for evaluating fetal cortical structures and brain volume [9]. Neurosonography is particularly used to assess the sulcification process in the Sylvian, calcarine, cingulate, and parieto-occipital fissures, and developmental delays in these fissures have been reported in FGR fetuses [10–13].

Recent studies have demonstrated that FGR is associated with delayed cortical maturation and reduced development of several brain structures, including the corpus callosum, cerebellar vermis, and cortical fissures such as the Sylvian, parieto-occipital, and calcarine fissures. These alterations appear to be more pronounced in cases with abnormal Doppler findings, suggesting a close relationship between hemodynamic impairment and delayed cortical development [11, 12, 14].

However, existing studies are heterogeneous, with inconsistencies arising from variable gestational ages at assessment and differing FGR definitions. Furthermore, many studies combine all fetuses below the 10th percentile (FGR + Small for Gestational Age (SGA)), conflating constitutionally small but healthy fetuses with true FGR cases [15, 16]. Additionally, while recent studies have shown relationships between Doppler parameters and structural brain changes, the impact of subclinical hemodynamic adaptation on cortical maturation remains insufficiently investigated [11, 12, 14].

This study aimed to evaluate cortical maturation and fissure development using neurosonography in fetuses with severe FGR (estimated fetal weight below the 3rd percentile) between 29 and 34 weeks of gestation. We compared these findings with those of gestational age-matched healthy controls.

Additionally, we investigated the relationship between cerebral hemodynamic adaptation (assessed by MCA PI) and fetal brain morphology.

## Methods

### Study design

This single-center, prospective case-control study was conducted between December 2024 and December 2025 at the Antalya Training and Research Hospital Perinatology Clinic. The study protocol was designed in accordance with STROBE (Strengthening the Reporting of Observational Studies in Epidemiology) guidelines. The study was approved by the Antalya Training and Research Hospital Clinical Research Ethics Committee (Approval no: 19/17, Date: 5.12.2024). Informed consent was obtained from all participants. The study adhered to the 2013 revision of the Declaration of Helsinki and Good Clinical Practice (GCP) guidelines.

### Group definitions and selection criteria

This study included singleton pregnancies between 29 + 0 to 34 + 0 weeks of gestation, where cortical sulcation accelerates, fissure depths can be reliably measured, and image quality is sufficient for neurosonography; the included pregnancies were referred to and subsequently followed in our department after the diagnosis of severe FGR and control. First-trimester ultrasound documentation was available for all cases, and gestational age was determined according to ISUOG recommendations using CRL measurements obtained between 11 + 0 and 13 + 6 weeks [17]. When first-trimester examinations had been performed externally, the available records were reviewed and verified prior to inclusion.

The FGR group consisted of fetuses with an estimated fetal weight (EFW) below the 3rd percentile. This definition aligns with the “severe FGR” subgroup identified by the Society for Maternal-Fetal Medicine (SMFM) and was chosen to exclude SGA cases that might be constitutionally small but healthy [1]. EFW was calculated using the Hadlock formula (incorporating biparietal diameter, head circumference, abdominal circumference, and femur length), and Intergrowth-21st reference curves were used for percentile assessment [18, 19]. UA PI and MCA PI percentiles were calculated according to Arduini and Rizzo [20]. Abnormal Doppler was defined as UA PI > 95th percentile and/or MCA PI <5th percentile. The cerebroplacental ratio (CPR) was calculated as MCA PI / UA PI; an abnormal CPR was defined as < 5th percentile according to Baschat and Gembruch [21].

The control group was selected from healthy singleton pregnancies attending routine antenatal care at our institution, with biometry between the 10th and 90th percentiles (AGA: appropriate for gestational age) and no detected fetal structural or placental anomalies. Following the identification of each FGR case, a consecutive AGA fetus meeting the matching criteria was prospectively enrolled as the corresponding control. Each FGR case was matched one-to-one with an AGA control fetus measured at the same gestational age (± 1 day). This matching methodology was designed to eliminate the strong influence of gestational age on cortical development as a confounding factor.

Multiple pregnancies; major fetal structural anomalies (including cardiac, renal, gastrointestinal, or skeletal anomalies); chromosomal abnormalities confirmed by amniocentesis or cordocentesis; suspected intrauterine infection indicated by positive TORCH serology; a history of maternal systemic disease (such as pregestational diabetes, autoimmune diseases, or chronic hypertension); hypertensive disorders of pregnancy (preeclampsia, gestational hypertension, or HELLP syndrome) developing during pregnancy; technically inadequate image quality due to fetal position or maternal obesity; and uncertainty regarding gestational age were excluded from the study.

### Obstetric and neurosonographic assessments

To eliminate operator variation, all imaging was performed by the same experienced physician (Perinatology specialist) using a Samsung Hera W10 (V8 platform) ultrasound machine with a 2–6 MHz convex transabdominal probe. In cases of cephalic presentation, an additional evaluation was performed using a transvaginal probe (4–8 MHz); in breech or transverse presentations, transabdominal evaluation alone was used. Transvaginal measurements were obtained in 58/82 FGR fetuses and 60/82 controls, whereas the remaining cases were evaluated using the transabdominal approach only.

Examinations were conducted in the multiplanar plane in accordance with the ISUOG “targeted neurosonography” Central Nervous System protocols [22]. All measurements were performed in the absence of fetal movement, with optimal image quality and standardized magnification settings. Each measurement was repeated three times, and the average was recorded. The coefficient of variation (CV) between measurements was controlled to remain below 5%.

Routine obstetric ultrasonography was performed in all cases. Fetal biometric measurements, including biparietal diameter (BPD), head circumference (HC), abdominal circumference (AC), and femur length (FL), were obtained using standard techniques. Estimated fetal weight (EFW) was calculated accordingly. In addition, fetoplacental Doppler parameters including the umbilical artery (UA), uterine artery, and MCA were evaluated, and ductus venosus Doppler assessment was performed when clinically indicated. Amniotic fluid volume and fetal heart rate were also recorded.

**Fig. 1:**
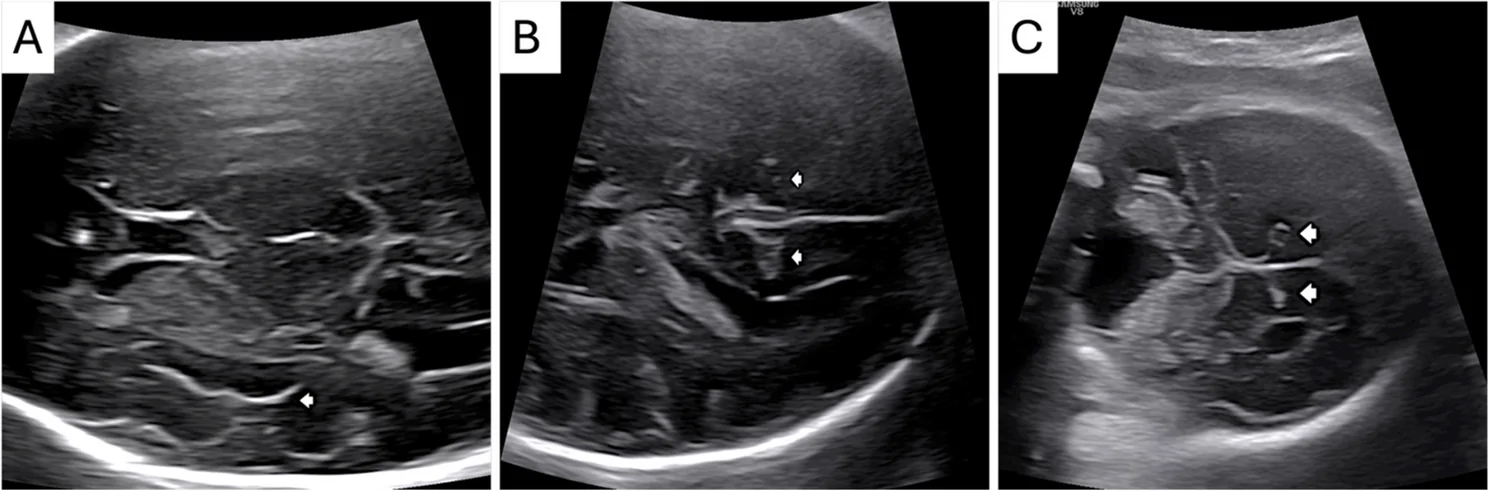
Neurosonographic assessment of cortical fissures. **A** Transventricular axial plane demonstrating measurement of Sylvian fissure depth (arrows) at 30 weeks and 2 days of gestation. **B** Transventricular axial plane showing the parieto-occipital fissure (arrow) in the same fetus. The fissure depth is measured perpendicularly from the midline to the apex of the fissure. **C** Coronal view through the occipital region demonstrating the calcarine fissure (arrows) in the same fetus. The depth is measured perpendicularly from the apex of the fissure to the midline. *All images were obtained using a Samsung Hera W10 ultrasound system with 2–6 MHz convex transabdominal probe at 30 weeks and 2 days of gestation*

**Fig. 2:**
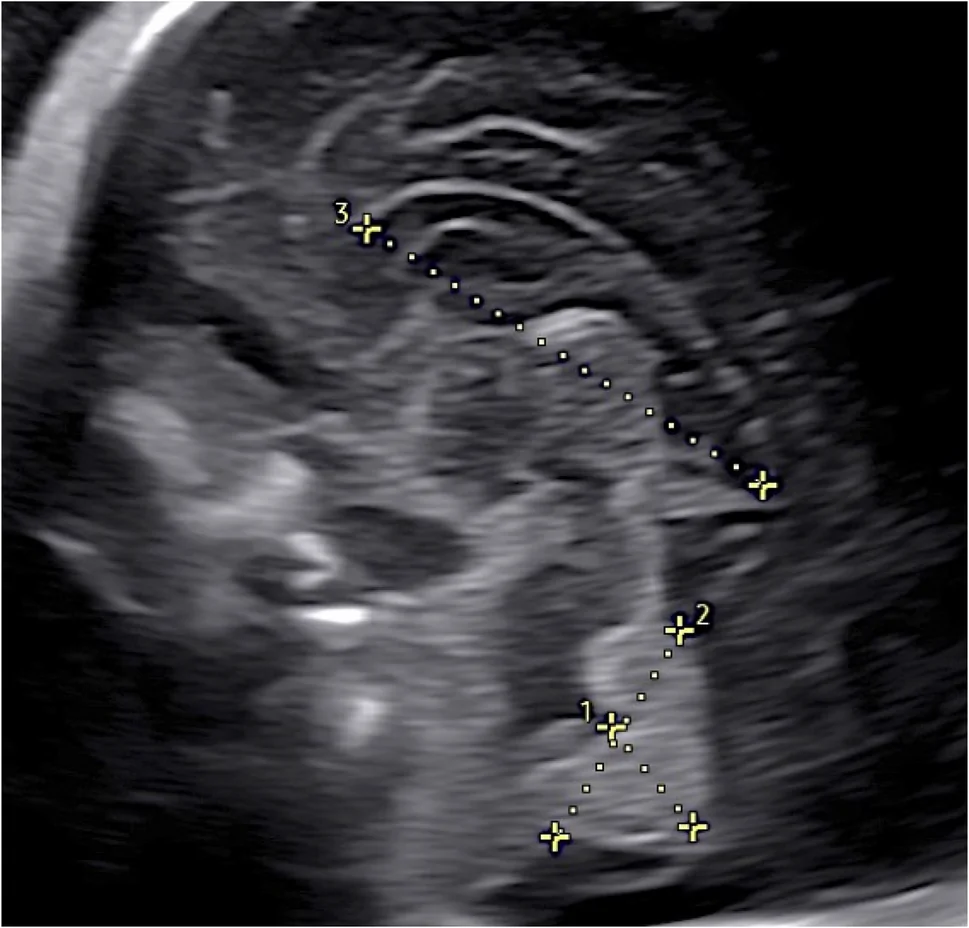
Grayscale ultrasound image in the midsagittal plane showing measurements of the corpus callosum (CC) and cerebellar vermis (CV). Dotted lines indicate the measurement locations

Fissure depth measurements and the additional neuroanatomical assessments were performed prospectively as part of the present research protocol and did not form part of routine clinical antenatal care. The Sylvian fissure (SF) was evaluated in the transventricular plane used for BPD measurement, where the cavum septi pellucidi, posterior horns of the lateral ventricles, and choroid plexus were visualized. A symmetric section was obtained with midline structures equidistant from the skull. SF depth was recorded as the “vertical depth” reflecting the operculization process, measured as the distance between the insular cortex and the inner layer of the parietal bone. Measurements were taken from the deepest point of the fissure and recorded in millimeters. The parieto-occipital fissure (POF) was assessed by slightly shifting the transducer cranially from the transventricular plane to obtain a triangular appearance. The POF was visualized in its characteristic Y or lambda shape. Depth was measured using a perpendicular line from the midline to the fissure apex and recorded in millimeters. The calcarine fissure (CF) was visualized in the occipital region in the coronal section obtained from the transcerebellar plane. Depth was measured by drawing a perpendicular line from the apex of the fissure to the midline and recorded in millimeters. (Fig. 1) The corpus callosum (CC) was evaluated in the mid-sagittal plane, where the cavum septi pellucidi, cavum vergae, brainstem, and fourth ventricle were simultaneously visualized. CC length was measured along a line from the most anterior point of the genu to the most posterior point of the splenium. The cerebellar vermis (CV) was also assessed in the mid-sagittal plane. Vermian length was defined as the maximal distance between the culmen and the uvula, while the anteroposterior vermian depth was measured as the perpendicular distance between the most anterior and posterior borders of the vermis. (Fig. 2) During measurement, the corpus callosum, brainstem, fourth ventricle, and cerebellar structures were aligned in the same image. CV length was defined as the maximum distance between the culmen (cranial) and the uvula (caudal). CV Anteroposterior depth was measured as the perpendicular distance from the most anterior point of the vermis to its most posterior point.

### Reproducibility of measurements

At the start of the study, intra-observer reliability was assessed in 20 cases (10 FGR, 10 control). The intraclass correlation coefficient (ICC) for measurements repeated by the same physician after one week was found to be > 0.85 for all parameters (excellent reliability).

### Statistical analysis

All data were recorded in Microsoft Excel 2019 using a double-entry method and checked for consistency. Statistical analyses were performed using IBM SPSS Statistics version 26.0 (IBM Corp., Armonk, NY, USA). The distribution of continuous variables was assessed using the Shapiro–Wilk test and further evaluated through histograms, Q–Q plots, and skewness–kurtosis values (acceptable range: −2 to + 2). Normally distributed variables were presented as mean ± standard deviation (SD), whereas non-normally distributed variables were reported as median with interquartile range (25th–75th percentile). Categorical variables were expressed as counts and percentages [n (%)]. Comparisons between groups were performed using the independent samples Student’s t-test for normally distributed continuous variables and the Mann–Whitney U test for non-normally distributed variables. Categorical variables were compared using the Chi-square test or Fisher’s exact test when the expected cell count was less than five. For statistically significant differences, effect size was calculated using Cohen’s d, interpreted as small (d = 0.2), medium (d = 0.5), and large (d = 0.8). Within the FGR group, correlations between hemodynamic Doppler parameters (MCA PI and umbilical artery pulsatility index [UA PI]) and neurosonographic measurements were evaluated using Pearson or Spearman correlation coefficients as appropriate. Correlation strength was interpreted as weak (|r| < 0.3), moderate (0.3 ≤ |r| < 0.7), or strong (|r| ≥ 0.7). Potential confounding by gestational age was minimized through a matched case–control study design. A two-sided p-value < 0.05 was considered statistically significant, and results were reported with 95% confidence intervals (95% CI).

### Sample size calculation

Sample size was calculated a priori based on previously published data on Sylvian fissure depth in late-onset FGR (Paules et al. [13]; AGA 11.75 ± 1.93 mm vs. FGR 10.89 ± 1.99 mm, Cohen’s d ≈ 0.44). To detect a between-group difference of this magnitude with α = 0.05 (two-sided) and 80% power, 82 participants per group were required. Accordingly, 82 FGR cases and 82 AGA controls were recruited. Power analysis was performed using G*Power 3.1.9.7.

## Results

### Study population characteristics

A total of 164 singleton pregnancies were evaluated, including 82 cases in the severe FGR group and 82 cases in the control group. As shown in Table 1, the groups were well matched with no statistically significant differences in maternal age (29.42 ± 5.75 vs. 28.82 ± 5.62 years; *p* = 0.522), body mass index (28.62 ± 5.26 vs. 29.18 ± 5.28 kg/m²; *p* = 0.566), gravidity (*p* = 0.853), parity (*p* = 0.947), or gestational age at evaluation (31.6 ± 1.6 vs. 31.5 ± 1.6 weeks; *p* = 0.722).

**Table 1:** Maternal characteristics and fetal biometric percentiles

	Control Group (*n* = 82)	FGR Group (*n* = 82)	*p*-value
Maternal Characteristics			
Maternal age (years)	28.82 ± 5.62	29.42 ± 5.75	0.522
Body mass index (kg/m²)	29.18 ± 5.28	28.62 ± 5.26	0.566
Gravidity	3 (1–3)	2 (1–4)	0.853
Parity	1 (0–2)	1 (0–2)	0.947
Maternal smoking, n (%)	7 (8.5)	10 (12.2)	0.610
Gestational age at evaluation (weeks)	31.5 ± 1.6	31.6 ± 1.6	0.722
Fetal characteristics			
Fetal sex, male/female	42/40	46/36	0.639
Fetal Biometric Percentiles			
BPD percentile	50 (11.5-84.75)	8.5 (3-32.25)	< 0.001
HC percentile	29 (13.75–52.5)	6 (1–13)	< 0.001
AC percentile	50 (25–69)	3 (1–3)	< 0.001
FL percentile	29 (17–46)	2 (1-3.75)	< 0.001
EFW percentile	35 (24.75-50)	3 (1–3)	< 0.001
Doppler Parameters			
UA S/D ratio	2.63 ± 0.44	3.29 ± 1.03	< 0.001
UA PI	0.93 ± 0.19	1.28 ± 0.47	< 0.001
MCA PI	2.10 (1.90–2.30)	1.75 (1.50–2.12)	< 0.001
CPR	2.35 ± 0.56	1.69 ± 0.76	< 0.001

Data presented as mean* ± *standard deviation or median (interquartile range)

*BPD *Biparietal diameter*, HC *Head circumference, *AC *Abdominal circumference,* FL *Femur length,* EFW *Estimated fetal weight,* UA *Umbilical artery,* PI *Pulsatility index,* MCA *Middle cerebral artery,* CPR *Cerebroplacental ratio

Fetal biometric percentiles were significantly lower in the FGR group compared to the control group across all parameters, including biparietal diameter (BPD), head circumference (HC), abdominal circumference (AC), femur length (FL), and estimated fetal weight (EFW) (all *p* < 0.001), confirming appropriate group assignment (Table 1).

Doppler velocimetry differed significantly between groups. The UA systolic/diastolic ratio (3.29 ± 1.03 vs. 2.63 ± 0.44; *p* < 0.001) and UA pulsatility index (1.28 ± 0.47 vs. 0.93 ± 0.19; *p* < 0.001) were significantly higher in the FGR group than in controls. Absent end-diastolic flow in the umbilical artery was observed in 3/82 FGR fetuses; no cases of reversed end-diastolic flow were identified. The MCA pulsatility index was significantly lower in the FGR group (median 1.75 vs. 2.10; *p* < 0.001). The cerebroplacental ratio was significantly lower in the FGR group (1.69 ± 0.76 vs. 2.35 ± 0.56; *p* < 0.001).

### Neurosonographic findings

**Fig. 3:**
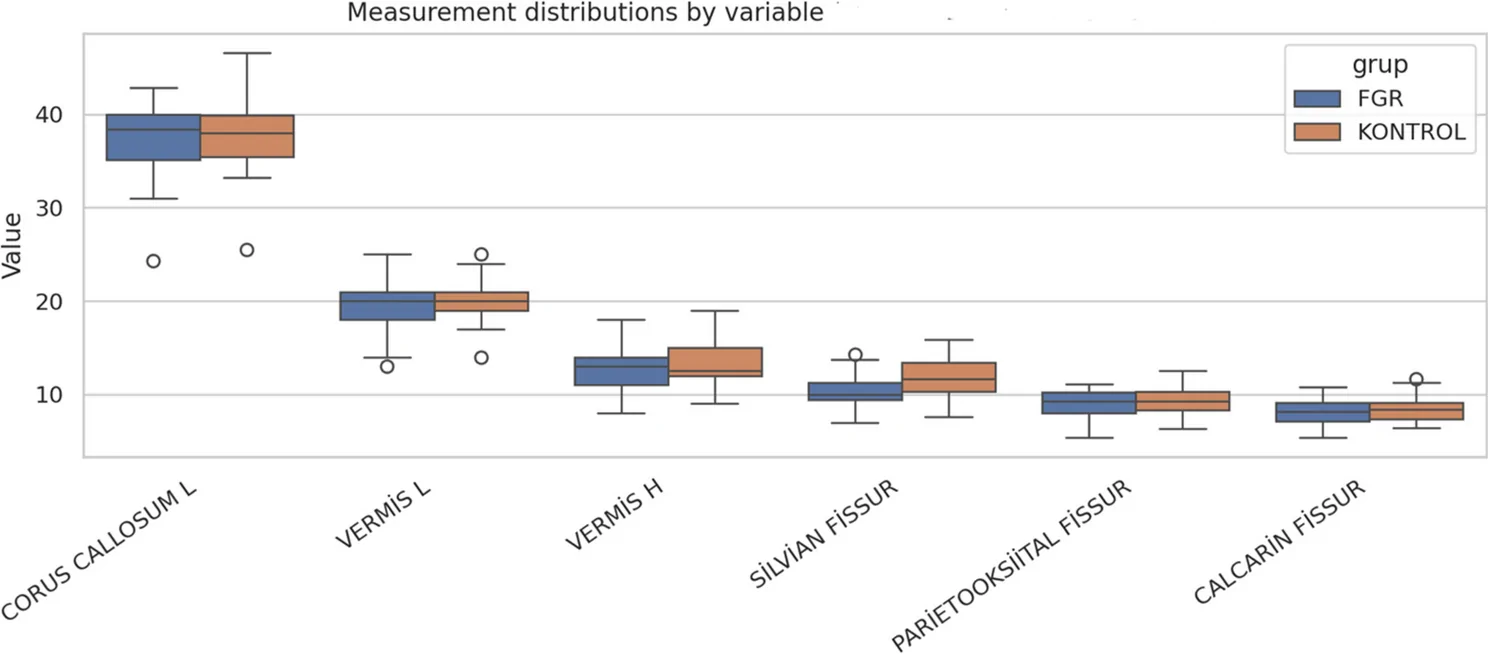
Distribution of fissure depth in Control and FGR groups

Regarding neurosonographic parameters (Table 2), Sylvian fissure depth was significantly shallower in the FGR group compared to controls (10.32 ± 1.71 mm vs. 11.79 ± 1.95 mm; *p* < 0.001). The difference in Sylvian fissure depth demonstrated a large effect size (Cohen’s d = 0.79), indicating a clinically meaningful difference between groups. Similarly, the calcarine fissure depth was significantly lower in the FGR group (8.07 ± 1.38 mm vs. 8.44 ± 1.24 mm; *p* = 0.043). No statistically significant differences were found for corpus callosum (CC) length, cerebellar vermis (CV) dimensions, or parieto-occipital fissure depth (all *p* > 0.05) (Fig. 3).

**Table 2:** Comparison of fetal neurosonographic parameters of the population

Neurosonographic Parameters	Control Group (*n* = 82)	FGR Group (*n* = 82)	Cohen’s d	*p*-value
Corpus callosum length (mm)	37.86 ± 3.74	37.29 ± 3.85	-0.15	0.331
CV length (mm)	19.62 ± 2.01	19.46 ± 2.61	-0.20	0.216
CV Anteroposterior depth (mm)	13.39 ± 2.83	12.95 ± 2.28	-0.17	0.297
Sylvian fissure depth (mm)	11.79 ± 1.95	10.32 ± 1.71	-0.79	**< 0.001**
Parieto-occipital fissure depth (mm)	9.24 ± 1.29	9.01 ± 1.44	-0.16	0.290
Calcarine fissure depth (mm)	8.44 ± 1.24	8.07 ± 1.38	-0.32	**0.043**
Data presented as mean ± standard deviation				

Bold values indicate statistically significant results (*p *< 0.05)

*CV *cerebellar vermis

In a subgroup analysis comparing FGR fetuses with brain-sparing (defined as MCA PI < 5th percentile) and without, no significant differences were observed in any of the neuroanatomical parameters (all *p* > 0.05). To explore the relationship between hemodynamic adaptation and brain morphology, correlation analyses were performed within the FGR cohort. Correlation analysis within the FGR group revealed significant inverse correlations between MCA PI and several neuroanatomical measurements (Table 3). A moderate inverse correlation was observed between MCA PI and calcarine fissure depth (*r* = -0.415; *p* < 0.001). Furthermore, weak-to-moderate inverse correlations were observed for CC length (*r* = -0.343; *p* = 0.003), CV length (*r* = -0.333; *p* = 0.004), and CV anteroposterior depth (*r* = -0.261; *p* = 0.027). No significant correlations were observed for Sylvian or parieto-occipital fissure depths in relation to MCA PI (*p* = 0.581 and *p* = 0.252, respectively). Furthermore, correlation analysis within the FGR group revealed that UA PI and CPR did not yield significant associations with any of the neuroanatomical measurements (all *p* > 0.05).

**Table 3:** Correlation Analysis between MCA PI and several neuroanatomical measurements

Neurosonographic Parameters	*r*	*p*-value
Corpus callosum length (mm)	-0.343	**0.003**
CV length (mm)	-0.333	**0.004**
CV Anteroposterior depth (mm)	-0.261	**0.027**
Sylvian fissure depth (mm)	-0.066	0.581
Parieto-occipital fissure depth (mm)	-0.137	0.252
Calcarine fissure depth (mm)	-0.415	**< 0.001**

Bold values indicate statistically significant results (*p* < 0.05)

*CV *cerebellar vermis

## Discussion

This study demonstrates that fetal brain morphology is significantly altered in fetuses with severe fetal growth restriction (FGR, <3rd percentile) compared with gestational age-matched healthy controls. Our primary findings indicate significant reductions in Sylvian and calcarine fissure depths in the FGR group. Notably, no differences were observed between Doppler-defined subgroups, whereas significant correlations with MCA PI were present.

The significantly lower SF depth observed in our FGR group (10.32 mm vs. 11.79 mm; *p* < 0.001) is consistent with recently published studies [5, 12, 23]. Mappa et al. reported that SF depth in fetuses with late onset FGR decreases progressively compared to SGA and AGA controls, with this reduction being more pronounced in the FGR group with abnormal Doppler findings [12]. These findings support and extend the early observations reported by Businelli et al. [5]. Ulusoy et al. also reported significantly lower SF depths in late onset FGR and noted that anterior cerebral artery (ACA) PI percentiles were significantly reduced [24]. This suggests that cortical fissural changes are associated with cerebrovascular adaptive changes detectable by neurosonography in both the MCA and ACA territories.

Similarly, the lower calcarine fissure (CF) depths detected in the FGR group (*p* = 0.043) indicate that development in the occipital region, where the visual cortex is located, is also affected by growth restriction. Mappa et al. reported that CF depth was significantly reduced in FGR, particularly in the group with Doppler abnormalities [12]. Alonso et al. demonstrated that CF grows linearly between 19 and 30 weeks of gestation in normal fetuses. The reduction observed in our FGR group, despite gestational age matching, suggests that CF development is directly affected by chronic hypoxemia [9]. In contrast, the lack of a significant difference in parieto-occipital fissure (POF) depth suggests that regional differences may exist in the brain’s sensitivity to hypoxia and the timing of maturation [6, 12]. While Mappa et al. found differences in POF depth between FGR and control groups, they noted that these differences varied according to Doppler status [12]. The absence of a significant difference in POF in our study may be related to sample size or the specific severity of FGR.

Reduced cortical fissure depths may serve as antenatal markers of later neurodevelopmental impairment affecting cognitive, motor, and language functions [2, 3]. The reduction in Sylvian fissure (SF) depth observed in our cohort (mean difference 1.47 mm; *p* < 0.001) should be interpreted in the context of normal fetal cortical development. Reference data indicate that the SF increases in depth at a rate of approximately 0.73 mm per week in healthy fetuses [9]; accordingly, the observed reduction corresponds to approximately two weeks of normal SF development. The SF is considered one of the most sensitive indicators of fetal cortical maturation, reflecting the process of opercularization, and its shallowing indicates delayed maturation of the insular cortex and the frontal and temporal lobes [8, 9]. Prior work has also linked comparable prenatal sonographic indices of SF and insular development to postnatal neurodevelopmental outcomes; in particular, Putra et al. demonstrated reduced normalized SF depth in fetuses with estimated fetal weight below the 10th percentile and reported that the prenatal hypoechoic insular zone circumference was inversely correlated with Bayley-III adaptive behavior scores at 2–3 years of age [25]. We emphasise, however, that our findings should be interpreted as a structural signal of altered cortical maturation rather than as direct evidence of altered neurodevelopmental outcome.

In a study evaluating fetal brain development between 31 and 35 weeks of gestation, no significant differences were observed in infratentorial and commissural structures such as the cerebellum, vermis, and corpus callosum [26]. Similarly, Yılmaz et al. also reported that these deep brain structures remained relatively preserved in fetuses with fetal growth restriction [23]. These findings suggest that cortical regions may be more sensitive to chronic hypoxia, whereas deeper brain structures remain relatively preserved. This pattern is consistent with our results, where significant reductions were observed in cortical fissure depths, while corpus callosum and vermian measurements remained unchanged between groups.

Within the FGR cohort, MCA PI values were negatively correlated with brain morphologic parameters, including calcarine fissure depth, corpus callosum length, and vermian dimensions. This relationship may reflect region-specific cortical remodeling associated with chronic fetal hemodynamic redistribution rather than true accelerated maturation. Our findings are consistent with the study of Ankara Aktas et al., who suggested that cortical development in FGR may demonstrate heterogeneous and region-specific neuroanatomical remodeling rather than uniform maturation delay [10].

CPR is widely considered a marker of fetal hemodynamic adaptation to placental insufficiency in FGR pregnancies, with reduced CPR values generally interpreted as reflecting redistribution of fetal cerebral blood flow in response to impaired placental function [27, 28]. In our study, CPR values were significantly lower in the FGR group, as expected; however, no significant correlations were observed between CPR and neuroanatomical measurements. Similar findings have also been reported by Ankara Aktaş et al., who did not identify significant associations between CPR and cranial structural parameters. These authors suggested that CPR may primarily reflect short-term fetal hemodynamic adaptation and the risk of intrapartum compromise, whereas cortical and midline brain structures may be more closely related to the cumulative structural effects of chronic hypoxia [10]. Accordingly, the absence of a direct association between CPR and fetal brain morphometry in our cohort may indicate that these parameters reflect different aspects and temporal phases of fetal cerebral adaptation.

From a clinical perspective, our results support the potential role of neurosonography in the assessment of neurodevelopment in FGR fetuses. Given the recognized association between FGR and adverse neurodevelopmental outcome, evaluation of fetal cortical development, particularly Sylvian fissure depth, may help identify fetuses with early brain remodeling associated with chronic placental insufficiency. However, given the absence of postnatal neurodevelopmental outcome data, these observations have been intentionally presented as exploratory and hypothesis-generating rather than as implications for immediate changes in clinical management.

One of the main strengths of our study is the inclusion of only severe FGR cases with an estimated fetal weight below the 3rd percentile, which allowed us to evaluate the relatively “pure” effect of uteroplacental insufficiency on fetal brain development while minimizing the potential bias introduced by constitutionally small fetuses. Furthermore, because fetal brain structures exhibit linear or exponential growth patterns across gestation, the strict one-to-one matching of the control and FGR groups with a precision of ± 1 day effectively eliminated gestational age as a confounding factor. This methodological approach strengthens the validity of our findings and supports the interpretation that the observed differences in neurosonographic measurements are directly attributable to growth restriction rather than maturational variation. Several limitations should be acknowledged. First, this single-center study did not correlate neurosonographic findings with postnatal neurodevelopmental outcomes. Second, neurosonographic measurements are inherently operator-dependent; because all measurements were performed by a single experienced clinician, inter-observer reliability could not be assessed, potentially limiting generalizability. In addition, the physician performing the measurements was not blinded to the clinical condition, which introduces the possibility of observer bias; to mitigate this, all measurements were performed in predefined standardised planes according to ISUOG neurosonography guidelines, each measurement was repeated three times with a coefficient of variation maintained below 5%, and the same protocol and equipment were applied to both groups. Third, uterine artery Doppler was not systematically recorded as part of the study protocol; future studies should include uterine artery indices to capture the maternal-side component of placental insufficiency. Fourth, matching was not performed for fetal sex; although no statistically significant difference in fetal sex distribution was observed between groups, this could not be excluded as a residual confounder. In addition, the study groups were not specifically matched with respect to imaging approach (transvaginal versus transabdominal ultrasonography). This methodological heterogeneity may have influenced the sensitivity and precision of neurosonographic measurements, particularly for subtle cortical parameters. Finally, the study population included only fetuses between 29 and 34 weeks of gestation. Therefore, the effects of growth restriction on cortical development at earlier or later gestational ages remain to be clarified in future multicenter studies with inter-operator reliability assessments.

## Conclusions

The findings of this study suggest that severe FGR is associated with selective alterations in prenatal cortical development, particularly involving the Sylvian and calcarine fissures, while cerebellar and corpus callosum structures appear relatively preserved. Within the FGR cohort, lower MCA PI values were associated with more pronounced morphologic differences in specific brain regions, suggesting a heterogeneous and region-specific pattern of cortical remodeling associated with chronic fetal hemodynamic redistribution rather than true accelerated maturation. Future prospective evaluation of the relationship between these antenatal findings and postnatal neurodevelopmental outcomes will contribute to the development of early diagnosis and intervention strategies for infants with FGR.

## Data Availability

The datasets generated and/or analyzed during the current study are available from the corresponding author on reasonable request.

## Abbreviations

FGR: Fetal growth restriction
MCA: Middle cerebral artery
PI: Pulsatility index
UA: Umbilical artery
EFW: Estimated fetal weight
CC: Corpus callosum
CV: Cerebellar vermis
SF: Sylvian fissure
POF: Parieto-occipital fissure
CF: Calcarine fissure
CPR: Cerebroplacental ratio
SGA: Small for gestational age
